# Psychosocial wellbeing and risk perception of older adults during COVID-19 pandemic in Nigeria: perspectives on the role of social workers

**DOI:** 10.3389/fpsyt.2024.1505279

**Published:** 2025-01-07

**Authors:** Farah Naz Rahman, Anthony Obinna Iwuagwu, Christopher Ndubuisi Ngwu, Michael Ebe Kalu, Amani Kasherwa, Anika Tasnim, Mohamman Rocky Khan Chowdhury, Mamunur Rashid, Manzur Kader

**Affiliations:** ^1^ Maternal and Child Health Division, International Centre for Diarrhoeal Disease Research Bangladesh (icddr,b), Dhaka, Bangladesh; ^2^ School of Public Health and Preventive Medicine, Monash University, Melbourne, VIC, Australia; ^3^ Department of Social Work, University of Nigeria, Nsukka, Nigeria; ^4^ School of Kinesiology and Health Science, York University, Toronto, ON, Canada; ^5^ School of Nursing, Midwifery, and Social Work, The University of Queensland, St Lucia, QLD, Australia; ^6^ Sidra Medicine, Ar-Rayyan, Doha, Qatar; ^7^ Unit of Public Health Science, Faculty of Health and Occupational Studies, University of Gävle, Gävle, Sweden; ^8^ Department of Medical Science, School of Health and Welfare, Dalarna University, Falun, Sweden

**Keywords:** older adults, loneliness, psychosocial wellbeing, risk perception, social work, COVID-19, Nigeria

## Abstract

**Background:**

The COVID-19 pandemic presented a ‘double-edged sword’ for older adults: not only were they more susceptible to the virus, but its broader consequences also exacerbated other challenges, particularly those related to psychosocial well-being. Limited evidence exists on how older adults perceive the pandemic and its impact on their well-being and the role of social workers in addressing these challenges, particularly in resource-limited settings like Nigeria.

**Aim:**

This study explored older adults’ perceived risks regarding COVID-19, its impact on their psychosocial well-being, and the role of social workers in addressing these challenges in Nigeria.

**Methods:**

A phenomenological and exploratory research design was used. In-depth interviews (IDIs) were conducted with 16 older adults and 4 social workers in Onitsha metropolis, Anambra State, Southeast Nigeria. Data were analyzed through reflexive thematic analysis.

**Results:**

The findings revealed that the COVID-19 restrictive measures negatively impacted the psychosocial well-being of older adults, where social isolation, lack of support, the inability to engage in wellbeing activities, and emotional trauma collectively contributed to a significant decline in their mental and emotional health. Additionally, widespread misconceptions about the origin of COVID-19 led to reluctance in adopting preventive measures. While social workers provided some awareness and counselling sessions, their involvement was limited. Social workers were not recognized as part of the frontline response team, and their efforts were primarily constrained by governmental and institutional neglect.

**Conclusion:**

The findings highlight the need for policy initiatives to enhance social workers involvement in strengthening the psychosocial resilience of older adults and addressing misconceptions during public health emergencies. Comprehensive strategies are essential for safeguarding the psychosocial well-being of older adults in future pandemics or similar crises.

## 1 Introduction

Loneliness, defined as the subjective feeling of being isolated, is a significant risk factor for poor health outcomes among older adults, such as functional disability, diminished cognitive functions, and poor quality of life (QoL) (1, 2), and in extreme cases, it can lead to depression and suicidal thoughts (3–6). Prior to the outbreak of the novel coronavirus (COVID-19) pandemic, loneliness was prevalent globally, particularly in Europe, America, and Asia (7, 8), and was termed a ‘behavioral epidemic’ (9) for older adults. The COVID-19 pandemic and its consequent restrictions have exacerbated these issues, especially for older people (10–12), who suffer a substantial proportion of mortality rates during the pandemic (World Health Organization [WHO], 2020).

The pandemic restriction measures in Nigeria, including lockdowns and social distancing, was implemented from mid-March 2020. While such measures are important to limit the spread of the infection, they might also have some negative implications for older adults (13, 14). These measures, aimed at curbing the virus’s spread, led to the closure of all sorts of public institutions and a total stay-at-home directive (15). Research indicates that COVID-19 transmission significantly rises during national and religious festivals (16), leading to stricter restrictions during these times, which are typically when nuclear families gather to spend time with their older relatives. Historically, Nigerian older adults living in intergenerational rural communities did not feel lonely due to frequent social gatherings and family visits (17). However, the pandemic’s restrictions on physical gatherings and travel heightened their social isolation and can potentially impact their psychosocial well-being, similar to observations in other countries (11). In developed nations, older adults maintain social connections during physical distancing through electronic devices like telephones and laptops. In contrast, older adults in resource-constrain settings like Nigeria face challenges due to limited access to such technologies and constraints from poor network services and high internet and call tariffs. This calls for a contextual exploration of the pandemic’s impact on the psychosocial well-being of older adults in limited resource settings like Nigeria.

In addition to the negative implications of social distancing measures, the pandemic has also been burdened by misinformation. In this digital age, many rely on social media for information, which oftentimes is unverified, misleading, and false (18). This misinformation significantly affects the psychological and physical well-being of people during crises like the COVID-19 pandemic. For example, the WHO reported that about 6,000 individuals were hospitalized, and at least 800 died globally in early 2020 due to misinformation spread by the media (19). Older adults, being the most susceptible to infection, are disproportionately affected by misinformation. Mukhtar (20) noted that lockdowns, physical distancing, and social media accelerate the spread of incorrect information, leading to detrimental effects on older adults. Studies have shown that people’s perceptions of the risk associated with COVID-19 influence their preventive practices (21–23). Evidence also suggests that older adults often display different and more conservative attitudes toward the pandemic and its preventive measures compared to other age groups (24, 25). Most of this evidence comes from a developed country perspective, and the sociocultural context of Nigerian older adults is substantially different. It is imperative to develop a contextual understanding of older people’s perceptions and attitudes toward the pandemic to devise a comprehensive preventive strategy for them.

Social workers in several countries played a significant role in reducing pandemic-related distress and misinformation (26). In more developed countries like the US and Italy, social workers served as front-line workers in the fight against COVID-19 (27). The International Federation of Social Workers [IFSW] (28) highlighted their roles during the pandemic, which included involving older adults in planning and response, providing mass education for COVID-19 prevention, and offering mental health services and alternative care for vulnerable groups like older adults. Evidence also suggests that interventions by social workers can reduce negative psychosocial consequences and improve knowledge on COVID-19 preventive behavior among older people (29–31). However, the specific roles of social workers in promoting the well-being of vulnerable groups, such as older adults, are rarely discussed in Nigeria. While one study by Isangha et al. (32) explored social workers’ roles and involvement during the COVID-19 pandemic in Nigeria, it was an opinion paper and did not incorporate the experiences and perspectives of social workers. As the pandemic impacted the work structure of social workers (33), it is important to explore their experiences and perceived roles in promoting older adults’ health during this crisis.

In light of the growing concerns around the impact of COVID-19 on older adults, particularly in terms of social isolation and misinformation, it is crucial to examine these issues in resource-constrain settings. Despite significant global research on the impact of COVID-19 on older adults, most studies have been conducted in developed countries, leaving a critical gap in understanding the unique experiences of older adults in low-resource settings like Nigeria. Additionally, while social workers globally played vital roles in mitigating these impacts, their specific contributions and challenges in Nigeria remain understudied. Although the COVID-19 pandemic has phased out, exploring its impact on Nigerian older adults remains crucial to inform future responses to similar crises. Understanding the psychosocial challenges faced by this vulnerable group during the pandemic can guide specific actions to mitigate these effects in future public health emergencies. Given the adverse experiences of older adults globally, examining Nigeria’s context strengthens the evidence base for addressing challenges in resource-constrained settings. Furthermore, evaluating the role of social workers during the pandemic is essential for improving policies and programs, ensuring their meaningful involvement in safeguarding the psychosocial well-being of older adults. Therefore, it is important to revisit the psychosocial impact of the pandemic on older adults and the subsequent role of social workers, which can contribute to generating evidence for resilience-building and better preparedness for future crises. This research, therefore, aims to fill the evidence gaps by exploring both older adults’ perceptions of COVID-19 and the pandemic’s impact on their psychosocial wellbeing, as well as the experiences of social workers supporting them. Understanding these dynamics will contribute to developing contextually appropriate interventions and preventive strategies for future public health crises.

## 2 Materials and methods

### 2.1 Study design

A phenomenological and exploratory research design was adopted to understand the perceived risk and psychosocial well-being of older adults, and the subsequent role of social work during COVID-19 by exploring the views of older adults and social workers. This design is particularly well-suited for this study because it focuses on capturing the lived experiences and subjective perceptions of individuals in response to a specific phenomenon—in this case, the impact of the COVID-19 pandemic on older adults. Phenomenological research allows for an in-depth understanding of how older adults and social workers interpret their experiences during a crisis, like the pandemic, thus providing qualitative insights that will help address the research objectives effectively.

### 2.2 Study site and participants

The study took place in Onitsha metropolis, Anambra State, Southeast Nigeria, which has a population estimated at 7,425,000 (34). Onitsha was severely impacted by the COVID-19 pandemic, and as of the time of data collection, the state had recorded 19 deaths from 909 cases (35). Being a high-risk zone, the lockdown, physical distancing and stay at home order of the government that were strictly in place in the state to prevent and curtail the spread of COVID-19 pandemic. One of the researchers was a resident in Onitsha throughout the period of lockdown and this informed the decision to conduct the study in this area.

The study involved In-depth Interviews (IDIs) with 16 older adult retirees, and Key Informants Interviews (KIIs) with 4 medical social workers. Inclusion criteria for older adult participants included being 60 years or older, residing in Anambra State, and being able to consent and communicate in English or Igbo. For social workers, inclusion criteria required having at least a postgraduate degree in social work and a minimum of two years of practical experience. A sample size of 20 was deemed sufficient for data saturation, as recommended for qualitative descriptive studies (36). It was also considered sufficient for a phenomenological study, as it allowed us to capture diverse perspectives and achieve thematic saturation. Qualitative research with phenomenological design prioritizes depth over breadth to explore lived experiences in detail, and established guidelines suggest that 10–15 participants are often adequate for phenomenological studies to achieve thematic saturation while maintaining manageable data complexity (36). There was an intention to increase the sample size if necessary; however, since data collection and analysis occurred concurrently, we determined that thematic saturation had been achieved with the current sample size where no new significant insights emerged from additional data. Additionally, the focus on a specific community with a relative homogeneity of experiences meant that fewer participants were needed to capture the essential themes. Feasibility concerns during data collection amid the pandemic further influenced the sample size, balancing practical constraints with the need for comprehensive and meaningful data. However, as a single-site study, we recognize the limitations in generalizability, and this has been acknowledged in the discussion section.

### 2.3 Sampling and data collection

We utilized purposive and snowball sampling techniques to recruit participants, while applying age and gender sensitivity in the selection to ensure a gender balanced view. Purposive sampling was well-suited for this study as it enables the selection of participants who possess specific characteristics or experiences relevant to the research objectives, thus ensuring that the insights gathered are meaningful and targeted. One of the researchers, a resident of the study area with a gerontological social work research background, utilized his local connections to recruit participants. Initially recruited participants also helped in identifying and recruiting additional respondents through the snowball sampling method. This strategy proved essential for reaching older adults who might have otherwise been difficult to engage due to the constraints posed by the COVID-19 pandemic.

We conducted this study during the first lockdown in Nigeria (April-May 2020), when the use of a physical distancing approach was essential in accordance with COVID-19 prevention protocols to ensure the safety of both the researchers and participants. Consequently, we adopted telephone interviews as a more suitable method for data collection. The use of electronic qualitative tools, such as telephones, has gained increasing relevance among researchers in recent years (37), particularly during the COVID-19 pandemic when safety measures were paramount. Interviews were conducted by two trained researchers (CN, AI) who are fluent in both English and Igbo. To address the limitations of telephone interviews, including the inability to observe non-verbal cues, researchers emphasized on rapport building and employed open-ended questions, probing questions, and active listening techniques to encourage participants to elaborate on their responses. The interviews, which lasted between 35-40 minutes, were semi-structured and conducted in both English and Igbo, depending on the participant’s language proficiency. A pilot study was conducted with two older adults and one medical social worker to refine the interview guide and determine the appropriate interview duration.

### 2.4 Data analysis

The data were transcribed and translated by two researchers (CN, AI) fluent in both languages, with an expert in Igbo linguistics reviewing the translations for accuracy. Two coders reviewed the transcripts line by line to generate initial codes and develop a working draft coding book, with sentences on COVID-19 perception and its impact on older adults, and the role of social workers as units of analysis. Although there was a working draft coding book, two coders continued to code line by line so that the original views of the respondents were retained. Reflexive Thematic Analysis (RTA) approach was adopted, where sub-themes were identified based on the nuances of participants’ experiences and were subsequently grouped into broader themes to inform the research objectives. This thematic analysis allowed the researchers to draw connections between the perceived risks and psychosocial consequences faced by older adults and the supportive roles undertaken by social workers during the pandemic. Although manual coding was employed, NVivo software was utilized to manage and organize the data analysis more efficiently. Peer member checking and reflective field notes were also employed to ensure trustworthiness and rigor throughout the research process.

## 3 Results

Respondents’ age ranged from 65 to 75 years among older adults, and 40 to 55 years among social workers. All the 16 older adults had attained at least a secondary level of education and were all civil service retirees, while the 4 key informants (social workers) all had a degree and still on active service in private and public sectors. Among the older adults, participants comprised of equal number of males [8] and females [8], while the key informants were all females [4]. See **Table 1** for full demographic description.

**Table 1:** Demographic characteristics of respondents.

Code	Age	Gender	Education	Occupation
001	67	Male	Degree	Retiree
002	66	Male	Degree	Retiree
003	69	Female	WASSCE	Retiree
004	65	Male	Degree	Retiree
005	65	Female	Degree	Retiree
006	69	Female	Degree	Retiree
007	68	Female	Degree	Retiree
008	75	Male	WASSCE	Retiree
009	70	Female	Degree	Retiree
010	65	Male	Degree	Retiree
011	68	Male	WASSCE	Retiree
012	71	Female	Degree	Retiree
013	67	Female	WASSCE	Retiree
014	69	Female	Degree	Retiree
015	65	Male	WASSCE	Retiree
016	65	Male	Degree	Retiree
017	40	Female	Degree	Social worker
018	55	Female	Degree	Social worker
019	41	Female	Degree	Social worker
020	40	Female	Degree	Social worker

WASSCE, West African senior secondary certificate examination.

Degree, Bachelor’s degree or its and other higher certificates obtained.

From the analysis of the interviews, a total of 10 subthemes emerged from various codes, contributing to the information on three overarching themes: Risk Perception of Older Adults Regarding COVID-19, Impact of COVID-19 on the Psychosocial Wellbeing of Older Adults, and Role of Social Workers in Supporting Older Adults During COVID-19 (**Table 1**). The following sections provide a detailed description of each theme.

### 3.1 Risk perception of older adults on COVID-19

**Figure 1:**
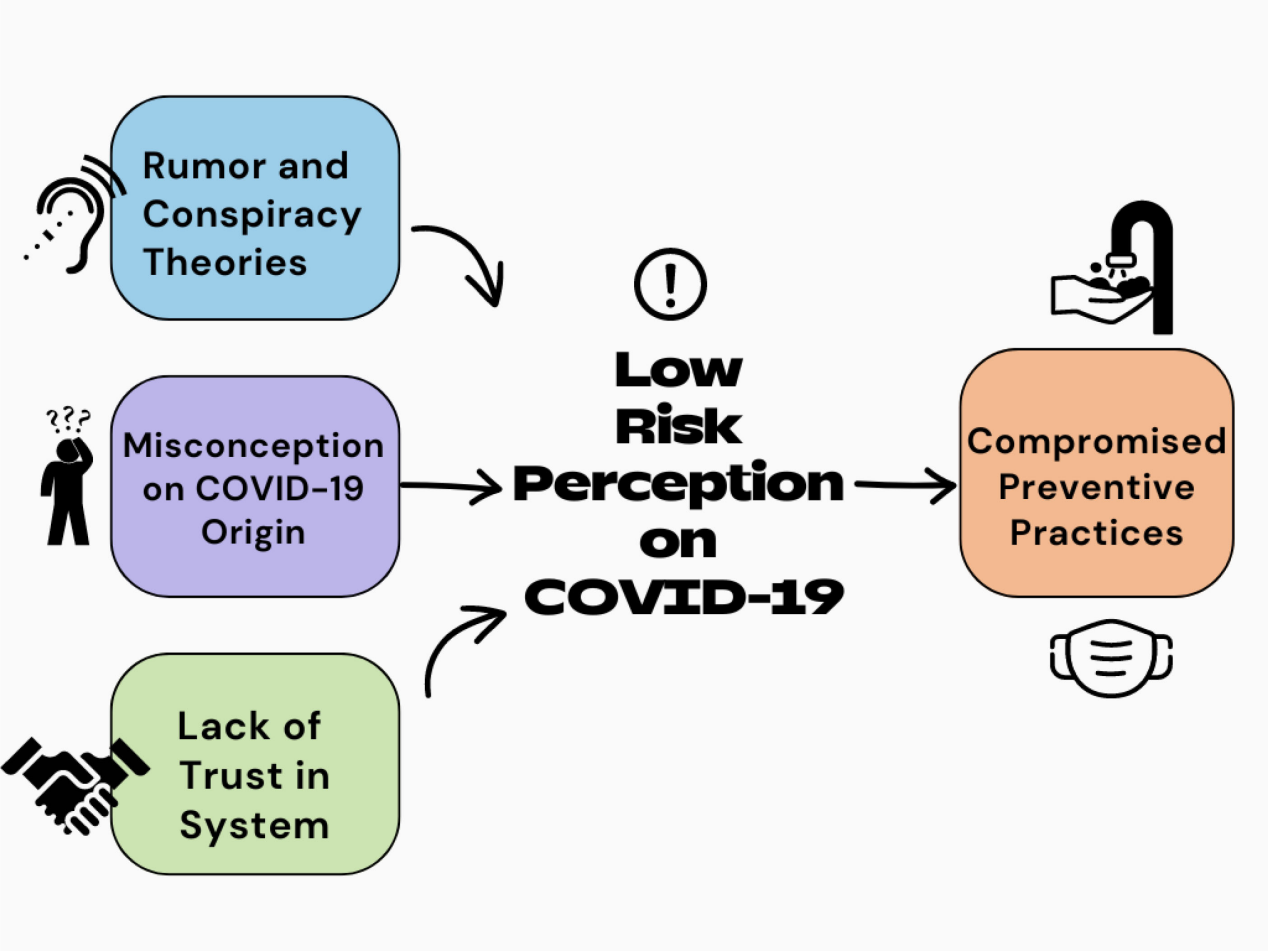
Interconnected subthemes on risk perception and subsequent preventive practices regarding COVID-19 among older adults in Nigeria.

The findings from this study suggest that there is a generally low risk perception among older adults regarding COVID-19. This low perception of risk is influenced by several factors, including the spread of rumors and conspiracy theories, religious and spiritual misconceptions about the virus, and a lack of trust in the government. These factors collectively impact how older adults perceive the threat of COVID-19 and, subsequently, influence their adherence to preventive measures (**Figure 1**). The findings were consistent across both genders.

#### 3.1.1 Rumors and conspiracy theories on COVID-19

A substantial number of participants believed in various conspiracy theories concerning the origin and intent behind the COVID-19 pandemic, which contributed to their belief that COVID-19 was either exaggerated or fabricated. These theories ranged from accusations against the Nigerian government to global plots by foreign nations. The common narrative among these older adults was the suspicion that COVID-19 was a government tactic to attract international funding, or that it was a strategic move by foreign powers like China to gain global supremacy. Such misinformation created confusion, leading to a dismissive attitude toward the virus, further lowering their risk perception.

“Till date, I still have some doubt if the virus exists at all because even the number of recorded cases and deaths in Anambra state is insignificant to what one should expect if the virus was truly here” [Older female:003].

“I have met people who argued that coronavirus does not exist and government is using the lockdown to make money from international agencies…” [Older male:001]

“Coronavirus was China’s strategy to gain economic advantage … and soon they started selling ideas and materials to the world to contain the virus” [Older female: 013].

#### 3.1.2 Misconception on COVID-19

Misconceptions about the virus were pervasive among the older participants, particularly those rooted in religious beliefs. For some, COVID-19 was interpreted as divine punishment for the moral failings of humanity. These spiritual explanations gave many older adults a false sense of security, believing that those who were “right with God” had nothing to fear from the virus.

“… this virus is strange; it must be a punishment from God. How else do we explain this plague to the whole world? The sins of the world have become too much … I am sure God is angry with the world. If you are at peace with your maker, then there is no need to be afraid, coronavirus is not for you [Older female: 014].

In addition to the religious angle, some participants believed that certain behaviors or conditions, such as alcohol consumption or smoking, could protect them from the virus.

“… there are rumors that Coronavirus cannot attack someone who smoke frequently because the virus cannot survive in a hit environment” [Older male:016].

Another participant added “if you take enough alcohol like I do, especially dry gin, you can be sure that coronavirus will stay far from you” [Older male: 010].

#### 3.1.3 Lack of trust in system

The lack of trust in the government also played a role in shaping participants’ risk perception. Some older adults were skeptical about the government’s handling of the pandemic, accusing it of politicizing the crisis. This suspicion reduced their willingness to comply with government-enforced lockdowns and other safety measures. Participants shared that they only adhered to COVID-19 restrictions, such as wearing face masks and observing social distancing, because they feared government penalties rather than the virus itself. They questioned the government’s motives and believed that the pandemic was being exploited for financial or political gain. This distrust diminished the effectiveness of public health messaging and made it difficult for older adults to take the pandemic seriously.

One participant noted, “No one knows if this whole coronavirus thing is true or not. I am saying this because everything in our country has been politicized. … I follow the rules because of the fines, not because I believe the virus is real” [Older male: 001].

#### 3.1.4 Compromised preventive practices

Older adults were reluctant to follow COVID-19 preventive measures, except when enforcement was strict. Many expressed dissatisfactions with the stringent measures imposed by the government, particularly the stay-at-home orders, social distancing rules, and the mandatory use of face masks. Compliance was often coerced through fines and penalties, rather than voluntary adherence based on an understanding of the risks. Even though these older adults were aware of the pandemic, their preventive practices were compromised due to their beliefs, misconceptions, and distrust.

One participant stated, “Left alone to me, I would not stay at home or maintain physical distance with anyone because I do not believe Coronavirus exists. I believe same applies to other people out there. You don’t expect a man to run away from what is not chasing him … however, he [Anambra State Governor] made sure people complied.” [Older female: 009]

Another shared an experience- “The COVID-19 task force asked me to pay 1,000 Naira (Nigerian currency) because I was exercising within my neighborhood without a face mask. After that ugly incidence, I don’t forget to use my mask outside my home.” [Older male: 006]

### 3.2 Impact of COVID-19 on the psychosocial wellbeing of older adults

The study findings suggest that COVID-19 and its consequences negatively impacted the emotional and social wellbeing of older adults in Nigeria. The pandemic triggered public health measures such as lockdowns, social distancing, and isolation, which, although necessary, had unintended and multi-faceted psychosocial consequences. Four interconnected subthemes emerged from the data: social isolation and loneliness, lack of social support, inability to engage in wellbeing activities, and emotional trauma and worry for the future (**Table 2**). Social isolation heightened feelings of loneliness and depression, while the lack of social support left many older adults feeling abandoned and rejected. The inability to participate in religious and social activities further deprived them of emotional and spiritual outlets, and the uncertainty of the pandemic, coupled with the loss of loved ones, contributed to emotional trauma and ongoing anxiety about the future. Together, these factors significantly shaped the overall psychological experience of older adults during the pandemic.

**Table 2:** Themes and subthemes of the phenomenological research on the psychosocial wellbeing and risk perception of older adults, and the subsequent role of social workers, during the COVID-19 pandemic in Nigeria.

Theme	Subtheme	Codes/Units
Risk Perception of Older Adults Regarding COVID-19	*Rumors and Conspiracy Theories*	- Belief in various conspiracy theories regarding COVID-19’s origin. - Suspicions that the pandemic is exaggerated or fabricated for political or financial gain
	*Misconception on COVID-19*	- Interpretation of COVID-19 as divine punishment. - Belief in behaviors (like smoking or drinking alcohol) as protective against the virus
	*Lack of Trust in System*	- Skepticism about government handling of the pandemic. - Compliance due to fear of penalties rather than belief in the virus’s threat.
	*Compromised Preventive Practices*	- Reluctance to follow preventive measures without enforcement. - Dissatisfaction with strict government measures.
Impact of COVID-19 on the Psychosocial Wellbeing of Older Adults	*Social Isolation and Loneliness*	- Heightened feelings of loneliness due to social distancing. - Emotional distress from loss of regular family visits
	*Lack of Social Support*	- Feelings of abandonment from family avoidance. - Loss of previously available emotional support.
	*Inability to Engage in Wellbeing Activities*	- Closure of community and religious activities disrupting emotional and spiritual fulfillment. - Loss of physical activities contributing to mental distress.
	*Emotional Trauma and Anxiety for the Future*	- Grief from losing loved ones and the inability to hold proper funerals. - Persistent anxiety about the future and fear for family safety.
Role of Social Workers in Supporting Older Adults During COVID-19	*Emotional Support and Counselling for Older Adults*	- Providing emotional support and reassurance through phone calls and visits. - Addressing anxiety and fear among older adults.
	*Dissemination of Accurate Health Information*	- Educating older adults about the virus and preventive measures. - Countering misinformation from social media and peers.
	*Facilitating Access to Social Services and Advocacy*	- Acting as intermediaries to connect older adults with essential services. - Advocating for older adults’ needs within local governments and organizations.
	*Minimal Involvement and Neglect from Government*	- Frustration over lack of recognition as essential workers. - Limited involvement in national COVID-19 response and relief activities.
	*Lack of Institutional and Private Sector Support*	- Challenges faced in private sector due to facility closures. - Institutional barriers limiting the ability to assist vulnerable older adults
	*Fear of Contamination and Exhaustion*	- Lack of access to protective equipment (PPE) - Increased risk due to vulnerable populations (older adults) - Health concerns for themselves and their families (e.g., parental duties) - Pre-existing stress and burnout from work

#### 3.2.1 Social isolation and loneliness

Most of the older adults expressed that the social distancing measures enforced to curb the spread of COVID-19 profoundly heightened feelings of loneliness and isolation among them. Majority of the participants described how they became physically isolated from family and friends, resulting in emotional loneliness, which eventually led to depressive symptoms such as fatigue, loss of appetite, and sleep disturbances. Before the pandemic, the older adults relied on frequent visits from family members, especially children and grandchildren, for emotional support and company. However, with the restriction of movement and fear of the virus, these visits ceased, leaving them in isolation. Their statements reflected the emotional toll of isolation, especially for older adults who were accustomed to regular family gatherings and in-person interactions with loved ones. This physical isolation also fostered a sense of abandonment and exacerbated feelings of loneliness, leading to mental distress.

One participant explained: “Staying at home all day without a single visitor or someone to interact with is the worse feeling you can give to an old woman like me. I get into deep thoughts often because I am alone. Most times, I start thinking and longing for my deceased husband. This gives me a whole new level of depression and keeps me delusional” [Older female:009].

Another participant reflected on how social distancing changed their regular family interactions: “My children always come around on weekends to stay with me. This lockdown and social distancing made it difficult to have my children and grandchildren around me and I cannot visit them either” [Older male:008].

#### 3.2.2 Lack of social support

Another important sub-theme that emerged was the lack of social support, which compounded the feelings of loneliness and emotional distress. The fear of infection led to social rejection, where older adults felt that even their closest family members avoided them due to fear of transmitting or contracting the virus. This further eroded the limited social support that was previously available to them.

One participant shared: “When I was able to meet with my son’s family, I practically felt like my daughter-in-law and my grandchildren were avoiding me. They acted like I was coronavirus itself. That is the worse feeling I have ever felt in my whole life” [Older male: 008].

This form of social rejection not only deepened feelings of loneliness but also created an emotional rift between the older adults and their families. Previously, many older adults found solace in their relationships with family members, but the pandemic disrupted this dynamic, leaving them without the emotional and social support they once had. The participants also spoke about their inability to connect with friends and neighbors due to lockdowns, contributing to an overall sense of isolation and loss of support.

#### 3.2.3 Inability to engage in wellbeing activities

Participants informed that a major source of wellbeing for many older adults in Nigeria comes from engaging in community and religious activities. They emphasized that these activities not only provide a sense of purpose but also offer emotional and spiritual fulfillment. However, due to COVID-19 restrictions, religious institutions and social gatherings were closed, leaving older adults without these vital outlets for social and emotional engagement. Some participants shared that the inability to attend religious services, which provided comfort and socialization, deeply affected their mental wellbeing.

One participant described the impact: “I used to go to church every Sunday and meet with my friends. Church was not just about praying, but also seeing people and feeling connected. With COVID-19, I can no longer go, and it makes me feel disconnected from the world” [Older female:012].

The closure of these communal spaces meant that older adults could no longer engage in the activities that nurtured their spiritual and mental health, leading to increased isolation and feelings of purposelessness. Some participants also lamented the inability to engage in physical wellbeing activities like group exercises, yoga, or even light outdoor walks, which had previously helped them manage physical pain and maintain emotional stability. The absence of such wellbeing activities diminished their emotional resilience, further contributing to their psychological distress.

One participant shared: “I can’t even go for my usual morning walks or join my yoga group. Staying indoors all day has worsened my joint pains, and now I feel mentally drained because I can’t do the things that used to keep me active” [Older male:014].

#### 3.2.4 Emotional trauma and anxiety for the future

The uncertainty surrounding the pandemic and the experience of losing loved ones created emotional trauma for some older adults. Participants expressed fear and anxiety about the future, with many grieving the loss of normalcy and the deaths of close friends or family members. For some, the inability to hold proper funeral rites for deceased loved ones due to the pandemic compounded their grief, leaving them with unresolved emotions and lingering trauma. This emotional trauma extended beyond grief for the deceased. Many older adults expressed a deep sense of worry about how long the pandemic would last and the uncertainty of what the future held. The persistent worry about the future, coupled with the trauma of loss and disruption, created an overwhelming psychological burden for older adults, many of whom felt helpless in the face of the pandemic.

One participant, who was unable to attend their mother’s funeral, reflected on the emotional burden: “Lockdown and social distancing was the reason I could not attend my mother’s burial at the village, and she was not celebrated accordingly due to the lockdown. Not being there to pay my last respect to my lovely mother has continued to give me sleepless nights” [Older male: 011].

The prolonged stress of the COVID-19 pandemic contributed to their declining mental health, as one participant illustrated: “I fear for my children’s safety, especially those living abroad. We get different right and wrong information about the virus, and this makes me anxious most of the time. My mind is always where they are, and I pray for them every day” [Older female: 012].

### 3.3 Role of social workers in supporting older adults during COVID-19

**Figure 2:**
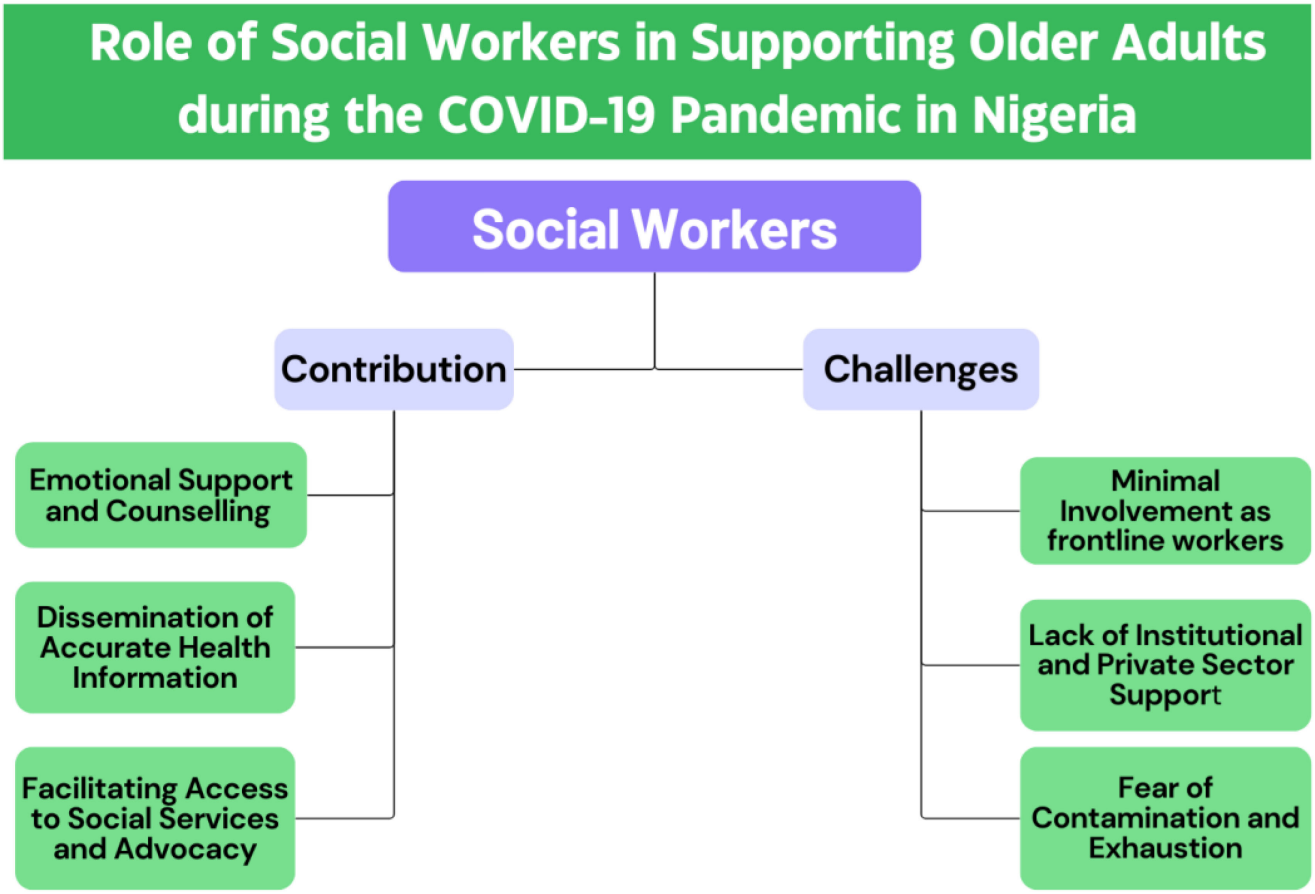
Subthemes related to the contribution and challenges of social workers in supporting older adults during the COVID-19 pandemic in Nigeria.

Social workers’ experiences revealed their contribution in supporting the psychosocial wellbeing of older adults, as well as the challenges they faced in performing their duties (**Figure 2**). We identified from the interviews of older adults and social workers that they provided essential services where possible, including offering counselling, raising awareness about the pandemic, and helping connect older adults to support facilities. However, as expressed by the social workers, their efforts were hindered by several challenges, such as minimal governmental engagement, lack of recognition as essential workers, institutional barriers, and lack of personal safety. The following sub-themes delve into both their contributions and the challenges encountered.

#### 3.3.1 Emotional support and counselling for older adults

In line with older adults, the social workers also emphasized that the psychosocial toll of the pandemic was evident in the heightened levels of anxiety, fear, and loneliness among older adults. From their experience, they shared that they tried to address these issues by providing emotional support and occasional counselling sessions, either through phone calls or in-person visits while maintaining COVID-19 safety protocols. They offered a listening ear to older adults who felt isolated due to lockdown measures and helped them process their fears related to the virus and the future.

One social worker noted: “Many older adults were scared and anxious, especially about the safety of their children and grandchildren, and their lack of touch with them. Our role was to listen, provide reassurance, and help them stay connected emotionally.”

An older adult commented on the value of these services: “The social worker who visited me helped me calm my fears. She would talk to me about how to stay safe and would always reassure me that things would get better. It made me feel less alone” [Older male: 014].

#### 3.3.2 Dissemination of accurate health information

Social workers played an important role in educating older adults on the nature of the virus, its transmission, and the importance of preventive measures such as wearing masks and social distancing. By sharing reliable information from credible sources, social workers contributed in improving older adults’ risk perception and encouraged them to follow safety guidelines. Social workers emphasized that providing access to reliable and accurate information was crucial in alleviating the anxiety and fears experienced by older adults, thereby reducing their psychological burden.

One social worker shared: “We had to constantly remind them that COVID-19 is real and dangerous, countering many of the myths they had heard from friends and on social media.”

#### 3.3.3 Facilitating access to social services and advocacy

Older participants shared that, with movement restrictions in place, many of them faced challenges in accessing essential services such as healthcare, food, and financial aid. From the experiences of older adults and social workers, it emerged that the social workers acted as intermediaries, helping older adults connect with these services, whether by organizing food distribution or arranging for home-based medical care. They also assisted with navigating bureaucratic processes for financial assistance programs, ensuring that older adults were not left unsupported during the pandemic.

One participant explained: “Our role was to ensure that no older adult went without food or medical care. We worked with local agencies to bring services directly to their homes.”

An older adult highlighted this role: “When I ran out of food, I didn’t know how to get help during the lockdown. I contacted one social worker through a helpline who arranged everything for me.” [Older male: 014].

Additionally, social workers also took on the role of advocates for older adults, ensuring that their unique needs and concerns were addressed by local governments and community organizations. They advocated for older adults to receive priority access to vaccines and mental health services and worked to raise awareness about the heightened risks this group faced. Additionally, social workers lobbied for more inclusive policies that would better protect older adults during future crises.

One social worker highlighted: “We had to speak up for older adults to ensure their needs were not forgotten in the rush to respond to the pandemic.”

Alongside their contributions, several challenges emerged from the experiences of social workers, which were further reinforced by the perspectives of older adults. Many older adults expressed that they had not seen as much involvement from social workers as they would have hoped during the pandemic. The following sub-themes provide context on these challenges and the barriers that limited social workers’ engagement.

#### 3.3.4 Minimal involvement and neglect from government

One of the key challenges identified was the minimal involvement of social workers in the national COVID-19 response. All social workers expressed frustration over being sidelined by government directives, which did not recognize them as essential workers. Many were mandated to stay home alongside other civil service workers, despite their potential to offer psychosocial support to vulnerable groups like older adults.

As one social worker lamented: “The government does not know the roles we are capable of playing … mainly medical staff was considered essential workers … but we could have helped the vulnerable cope with life during the pandemic” [Social worker: 020].

Some social workers took it upon themselves to offer voluntary services outside their official duties, but these efforts were limited. The government’s failure to include social workers in pandemic relief activities, such as palliative distribution, further hindered their ability to assist older adults in need. Participants expressed that this lack of institutional recognition significantly curtailed their contributions during a critical time.

One participant informed, “I even heard that some social workers who work in the ministry of women and children affairs were not allowed to be involved in palliative distribution … neglecting social workers” [Social worker: 017].

#### 3.3.5 Lack of institutional and private sector support

Social workers also encountered challenges related to the institutions they worked for, especially in the private sector. Some facilities, particularly private medical institutions, closed down or reduced operations due to fear of the virus and inadequate resources to manage COVID-19 patients. As a result, many social workers were sent home without pay, leaving them unable to fulfill their roles.

One social worker from the private sector explained: “The private medical facility I work with went on a total lockdown … the management ran out of funds to keep me as their social welfare worker. I was asked to go home until things normalize” [Social worker: 019].

It was emphasized by the participants that this lack of support from both the government and private institutions not only impacted the livelihood of social workers but also hampered their ability to provide much-needed psychosocial care to older adults.

#### 3.3.6 Fear of contamination and exhaustion

Fear of contamination was another significant barrier to social workers’ involvement during the pandemic. Many social workers, particularly those in older age groups, were concerned about their own health and the risks associated with working directly with vulnerable populations. This fear was exacerbated by the severity of the virus among older adults, and the lack of access to protective equipment.

As one social worker shared: “I have parental duties too, and my children expect me to be safe for them. I don’t want to go out and expose myself without proper protective equipment” [Social worker: 018].

This fear, coupled with insufficient protective measures for social workers, further reduced their willingness to engage in direct support for older adults, particularly in high-risk environments such as hospitals and care facilities.

Exhaustion and burnout also emerged as challenges for social workers, many of whom were already overworked before the pandemic. When the stay-at-home orders were implemented, some social workers expressed relief, viewing it as an opportunity to recover from work-related stress. This exhaustion limited their ability to provide consistent and proactive support to older adults during the crisis.

One social worker noted: “… before the outbreak of coronavirus, I had over labored myself at the workplace and was feeling stressed out … I was excited because I needed the rest so much” [Social worker: 017].

## 4 Discussion

This study offers important insights into older adults’ perceptions of COVID-19, its psychosocial impact, and the role of social workers in supporting them during lockdown in Nigeria. The study found that varied views and misconceptions about the origin, spread and prevention of COVID-19 pandemic exist among older adults in Anambra state Nigeria. The older adults however complied with the prevention measures such as lockdown and stay at home even though it was not their free will. The perceived risk for COVID-19 was generally low risk among older adults. This low-risk perception influenced by conspiracy theories, religious beliefs, distrust in the government, and misconceptions about COVID-19, played a crucial role in shaping their attitudes and behaviors toward preventive practices. This complex web of influences can potentially make it challenging for public health initiatives to effectively engage and protect this vulnerable demographic in future crises. The study also discovered that COVID-19 pandemic preventive measures such as lockdown and social distancing orders has negatively impacted the psychosocial wellbeing of older adults. The psychosocial impact of COVID-19 on older adults in Nigeria was multifaceted. Social isolation, lack of support, the inability to engage in wellbeing activities, and emotional trauma collectively contributed to a significant decline in their mental and emotional health. The pandemic restrictions, although necessary, heightened their vulnerability, leaving them grappling with feelings of loneliness, rejection, and anxiety. This calls for strategies to addressing the psychosocial needs of older adults in mitigating the long-term mental health consequences during the future waves of the pandemic or in similar crises. Moreover, this study uncovered those social workers were not regarded as frontline workers during first lockdown in Nigeria and some contributed only as volunteers in the fight against COVID-19. While social workers in Nigeria did play a role in supporting the psychosocial wellbeing of older adults during the pandemic, their efforts were constrained by governmental and institutional neglect, fear of contamination, and pre-existing burnout. These challenges underscore the need for a more structured and supportive framework for social workers in public health crises, especially in ensuring the mental and emotional wellbeing of vulnerable populations like older adults.

Regarding risk perception, the study revealed that all the participants were aware of coronavirus but they hold varied views, misconceptions, assumptions and conspiracy theories about the origin and nature of the virus. Among the conspiracy theories and assumptions older adults have on COVID-19 are: The virus does not exist at all; it is a punishment from God and cannot infect anyone who has not offended God; it is a Chinese made virus to gain power; there are political inclinations to the outbreak etc.; Alcohol and smoking prevents infection of COVID-19 etc. Kazeem (38) is in agreement with this finding when he confirmed that social media have been used negatively to spread fake and unconfirmed news about the virus, instilling fears and confusion in people. According to Kaur (39), CNN have also taken some steps to clarify some of these baseless myths people hold about the virus and WHO has separated itself from the wrong preventive assumption propagating social media. For example, CNN debunked that black people are immune to coronavirus, smoking and consuming alcohol keeps one immune to the virus and children cannot be affected by the virus etc. Supporting this finding and taking one step further, the African zone of World Health Organization conducted workshops on COVID-19 to debunk false claims about the virus and equally sensitized Nigerians on the evidence-based COVID-19 preventive measures and the need for strict compliance (19). Again, it was found that respondents reluctantly complied with the COVID-19 lockdown orders especially stay at home and social distancing. We found that such compliance did not come out of old people’s intention to prevent the virus or to comply with COVID-19 guidelines but for their personal interests to escape punishment melted on defaulters. Another earlier study in Nigeria on COVID-19 knowledge and practice among general population supports this finding by mentioning that hand washing among people was usually undertaken as a traditional practice and not as a preventive measure for COVID-19. In line with our findings, several previous studies also concluded that lower perceived risk of the virus contributes to reluctance in the practice of preventive measures (21–23).

In addition, the study found that COVID-19 pandemic had negative impact on older adult’s psychosocial health as they were always afraid for their children and grandchildren who live within Nigeria and in Diaspora. The propagation of different news and conspiracy theories on COVID-19 did not spare the emotional health of older adults too as they often felt anxious over the opposing information they get about the virus. Worst still, the stay-at-home measure created loneliness among older adults and this worsened their mental health as they reported extreme depression and delusion caused by prolonged physical distancing and loneliness. In agreement with this finding, studies have reported the association of mental health issues like suicide and depression to loneliness among older adults (3, 5, 6) keeping them at risk. A couple of literature reviews, supporting our findings, also reported that the COVID-19 restrictive measures increase the loneliness and subsequent mental health issues among older adults (10, 11). Several studies in China have reported similar findings, highlighting significant mental health challenges during the COVID-19 pandemic (40–42). Loneliness was prevalent among older Chinese adults, while a sense of alienation was directly associated with depression and poor sleep quality (40, 41). The pandemic had an unprecedented mental health impact on the general population as well, with many seeking internet-based mental health services (42). These findings underscore the critical importance of public health research and interventions to address psychosocial and mental health needs during pandemics. In resource-constrained settings, social workers can play a pivotal role in addressing these challenges and providing targeted support during crises.

Lastly, our findings show that majority of the social workers were not actively involved in professional engagement during COVID-19 pandemic lockdown. The few who were involved acted as volunteers as their job or government did not recognize them. This contradicts findings of other studies that reported adequate involvement of social workers in combating COVID-19 pandemic in different part of the world where they were frontline workers (43–47). This variance in finding perhaps could be attributed to study settings (United Kingdom, New Zealand, Italy, South Africa; China) where social work is institutionalized and welfare is of greater priority compared to Nigeria where we conducted the study. The International Federation of Social Work (28), further buttressed the participation and roles of social workers during COVID-19 pandemic thus: “In many countries social workers are supporting communities that are affected or fearful of the COVID-19 virus”.

On the normal, social workers during a pandemic function as social planner, community organizer, advocate, teacher, facilitator, broker amongst other roles. Even more important to the present study are roles such as community education for, counseling services and psychotherapy. This will help reduce the mental health effect of loneliness, suicide and depression on older people as well as ensure that older people as a vulnerable group adheres to preventive measures of COVID-19 and are re-orientated about the virus and need for vaccine intake. They could as well advocate for social services and facilitate community or group programs to strengthen the mental well-being of older adults. Lastly, social workers are responsive to the overall wellbeing of old people. They take into cognizance, the effects of social distancing and the psychology of adapting to a changed environment as part of their intervention with older people in an epidemic or pandemic situation like this. Another study on COVID-19 in Nigeria (39) also recommended that social workers should work side by side medical doctors as members of a multidisciplinary team for the achievement of the overall wellbeing of older adults. Indeed, there are many roles for social workers in a pandemic ravaged society and their intervention is much needed.

Given the scarcity of social workers in the fight against COVID-19 in Nigeria and the poor governmental recognition as a factor found in this study, it is our recommendation that the Nigerian government should pay better attention to the profession of social work, pass the social work bill into law, and give full credence and support for professional practice in the country. In the interim, we recommend that more social workers should uphold their professional mandate of service to humanity by offering volunteering services to agencies taking care of old people who are susceptible to mental health risks occasioned by the COVID-19 pandemic, lockdown and social distancing measures.

## 5 Strengths and limitations

This study sheds light on an under-researched area by exploring the role of social workers and the experiences of older adults during COVID-19 in resource-limited settings, providing a merged view from both perspectives. Although the study produced significant results with important implications for social work practice and future research, several limitations should be acknowledged. The findings reflect the views of a small subset of older adults from South Eastern Nigeria, limiting the diversity of perspectives, particularly from older adults in rural areas and other regions of the country. As a result, the data may not fully capture the broader experiences and risk perceptions of older adults across different socio-economic and geographical contexts. Additionally, the small sample size of both older adults and social workers restricts the study’s ability to generalize the findings. Future studies should aim for a larger, more diverse sample across multiple regions to ensure a more comprehensive understanding of the psychosocial impacts of COVID-19 and the role of social workers in supporting older adults. Furthermore, the study relied on self-reported data, which may be subject to recall bias or social desirability bias, and did not include longitudinal follow-up to assess longer-term effects. Expanding the scope of future research to include longitudinal designs and mixed-methods approaches could provide deeper insights into these critical issues.

## 6 Conclusion

Considering the findings discussed above, it is evident that the COVID-19 pandemic has had profound impact on the older adult population in Nigeria. Nigerian older adults have experienced social isolation rather than mere physical distancing, which has heightened their feelings of loneliness. Many older adults hold misconceptions about the origin and existence of the COVID-19 pandemic, leading to a lack of adequate information that contributes to low-risk perception and compromises their compliance with preventive practices. Consequently, this misunderstanding has resulted in reluctance to follow guidelines and protect themselves effectively. The division among older adults regarding their willingness to comply with preventive measures underscores the need for targeted awareness efforts. Nigerian social workers were largely active as volunteers during the initial lockdown of the COVID-19 pandemic, yet their contributions were hindered by a lack of government recognition. Therefore, the study recommends that for future public health crises, the knowledge and awareness of older adults, as well as their psychosocial health, should be addressed through comprehensive strategies involving social workers to ensure better compliance with health guidelines and support their overall wellbeing.

## Data Availability

The raw data supporting the conclusions of this article will be made available by the authors, without undue reservation.
