# Supplementary material for: Psychosocial wellbeing and risk perception of older adults during COVID-19 pandemic in Nigeria: perspectives on the role of social workers
Source: Front Psychiatry. 2025 Jan 7;15:1505279. doi: 10.3389/fpsyt.2024.1505279 (PMC11747111; doi:10.3389/fpsyt.2024.1505279)
Supplement: Supplementary file 1 [file Table1.docx]

**Supplementary Material**

Supplementary Table 1. Interview guide of older adults and social workers for the phenomenological research on the psychosocial wellbeing and risk perception of older adults, and the subsequent role of social workers, during the COVID-19 pandemic in Nigeria

| Interview guide for older adults to describe their perception of COVID-19, their experiences during the COVID-19 lockdown, and experiences with social workers | Interview guide for social workers to describe their experiences and expectations during first lockdown |
| --- | --- |
| a. What are your views on COVID-19 pandemic?   - Probe for the origin/knowledge of COVID-19 - Probe for preventive measures - Probe for lockdown   b. What are your views on the compliance of COVID-19 prevention protocols?   - Why is it? - Why not?   c. In what ways have you been affected by the lockdown and distancing orders   - Probe for impact of social distancing orders - Probe for impact of lockdown orders - Probe for loneliness - Probe for psychological impact   d. What are your experiences with social workers during the COVID-19 lockdown?   - Why is it? - Why not? | 1. How would you describe your role in supporting older adults during the COVID-19 pandemic?  - Probe for involvement - Probe for lack of involvement - Probe for psychosocial support - Probe for awareness  1. What are the factors impacting social workers’ role during the COVID-19 pandemic in Nigeria?  - Probe for governmental factors - Probe for legislative reasons - Probe for personal reasons - Probe for other emerging factors  1. Please share your expectations or recommendations for social workers role during COVID-19  - Probe for involvement in gerontology social work |
| Thank you for participating | |
